# Conjugation of visual enhancers in lateral flow immunoassay for rapid forensic analysis: A critical review

**DOI:** 10.1007/s00216-024-05565-6

**Published:** 2024-10-09

**Authors:** Maria Dede, Annemieke van Dam

**Affiliations:** 1 Department Biomedical Engineering & Physics, Amsterdam UMC, University of Amsterdam, Meibergdreef 9, Amsterdam, 1105 AZ Netherlands; 2 Department Forensic Science, Amsterdam University of Applied Sciences, Tafelbergweg 51, Amsterdam, 1105 BD Netherlands; 3 Methodology Research Program, Amsterdam Public Health Research Institute, Amsterdam UMC, Meibergdreef 9, Amsterdam, 1105 AZ Netherlands

**Keywords:** Visual enhancers, LFA, Conjugation, Forensic, Nanoparticles

## Abstract

During crime scene investigations, numerous traces are secured and may be used as evidence for the evaluation of source and/or activity level propositions. The rapid chemical analysis of a biological trace enables the identification of body fluids and can provide significant donor profiling information, including age, sex, drug abuse, and lifestyle. Such information can be used to provide new leads, exclude from, or restrict the list of possible suspects during the investigative phase. This paper reviews the state-of-the-art labelling techniques to identify the most suitable visual enhancer to be implemented in a lateral flow immunoassay setup for the purpose of trace identification and/or donor profiling. Upon comparison, and with reference to the strengths and limitations of each label, the simplistic one-step analysis of noncompetitive lateral flow immunoassay (LFA) together with the implementation of carbon nanoparticles (CNPs) as visual enhancers is proposed for a sensitive, accurate, and reproducible in situ trace analysis. This approach is versatile and stable over different environmental conditions and external stimuli. The findings of the present comparative analysis may have important implications for future forensic practice. The selection of an appropriate enhancer is crucial for a well-designed LFA that can be implemented at the crime scene for a time- and cost-efficient investigation.

## Introduction

Biological traces (e.g., blood, urine, semen, vaginal fluid) and fingermarks are valuable pieces of evidence that can be found on objects or surfaces at the crime scene. The collection and analysis of such traces may be proven crucial for the overall case investigation. Traditionally, biological fluids are collected and further submitted for short tandem repeat (STR) DNA analysis. If fingermark traces are recovered, these are currently analyzed by carefully inspecting the ridge pattern (e.g., loops, arches, and whorls) or by extracting the DNA and performing profiling analysis to individualize the donor [1–3]. However, such methods are optimal when the traces collected bear sufficient DNA quantity and adequate quality for STR profiling, and when “ideal” fingermarks are found, namely complete, abundant in DNA, and not contaminated [4]. Therefore, these traditional analytical techniques show important limitations when implemented for complex and/or sub-optimal trace examination [3, 5].

Beyond DNA profiling and examination of fingermark impressions left on a surface upon contact, potentially relevant chemical information may be obtained that are currently disregarded. In the investigative phase, when the perpetrator is unknown, the examination of the chemical composition of a trace may provide crucial intelligence information and assist in the profiling of the donor. More precisely, information concerning the age and sex of the donor, disease-status, potential drug consumption, or manipulation of illicit substances and/or explosives may be obtained [4, 6, 7]. Furthermore, in contrast to DNA profiling, there are barely any legal implications for the recovery and use of chemical information from crime scene traces making such analytical approach potentially applicable in the future [4, 8, 9]. However, the endorsement and utilization of the chemical profiling analysis depend on the specific legislation of each country, which determines its acceptance and application within the legal system [10]. Lastly, based on the objectives of the investigation, this analytical method exhibits versatility. The chemical compounds that are usually targeted and analyzed can be classified under two main categories, namely, endogenous (e.g., proteins, lipids, (drug) metabolites) or exogenous (e.g., drugs, cosmetics, explosives, dust) [4, 7, 11–13].

One promising technique for the chemical profiling analysis of biological traces in routine forensic casework is the lateral flow immunoassay (LFA). LFA is a well-validated and widely used technique in the biomedical, food, and agricultural fields that has been gaining interest among the forensic research community for body fluid identification at the crime scene [14–16]. The LFA is more suitable for the rapid detection of analytes rather than sophisticated analytical methods like liquid chromatography-mass spectrometry (LC–MS) [15]. The implementation of LFA appears promising mainly due to its simplistic setup and the way traces are analyzed [17]. The small microfluidic device consists of a nitrocellulose membrane that bears the strips where capture molecules are immobilized to detect and bind the target compounds present in the investigated sample. Depending on the type of visual enhancer that is used, a color change or a fluorescence emission will signal the presence of the compound in the examined trace [18]. The specific compound or substance under investigation is identified as the analyte. In the forensic context, important requirements for the implementation of the LFA are sensitivity, specificity, and reproducibility of the results. The results obtained by LFA must, therefore, be of high quality, minimizing the risks of false negative and positive outcomes. For this reason, researchers focus on the implementation of different visual enhancers and conjugation techniques to optimize the sensitivity, reducing the detection limits of the LFA systems [19].

The present review provides a detailed overview of the state-of-the-art visual enhancers implemented throughout the different research fields. A series of criteria relevant to the forensic practice are taken into consideration to evaluate the different visual enhancers. The criteria selected for assessing the effectiveness of a LFA setup included sensitivity, reproducibility, visualization method, costs, size, enhancement potential, and type of signal. An efficient LFA must adhere to these stringent criteria to be deemed suitable for implementation in forensic practices. Upon comparison, the most suitable visual enhancer for use in LFA setups during routine forensic casework for the purpose of trace identification, donor profiling, and/or individualization has been determined.

## The principle of LFA

The LFA apparatus consists of multiple components each with a specific role to guarantee a fast and efficient analysis. More precisely, a sample application pad allows for the transfer of the sample from the insertion point throughout the membrane via capillary action; the conjugation pad serving as a reservoir for the detection antibodies conjugated to the visual enhancers; the nitrocellulose membrane where the capture compounds are fixed on the strip and ready to recognize the analytes through specific binding domains; the absorbent pad that removes the excess buffer on the nitrocellulose strip thus maintaining a stable flow rate and enabling the accommodation of larger sample volumes; and the backing pad for supporting the aforementioned components [19]. The use of LFAs has rapidly increased mainly due to the low costs and their user-friendly nature, making their manipulation by non-trained personnel at the crime scene feasible. The LFA layout and the analytical procedure that is followed render this technique time efficient, completing the analysis within a timeframe of approximately 10 min [14, 20]. This one-step analysis can potentially yield crucial qualitative and quantitative results. Furthermore, the stability and the minimized impact of external conditions make this technique portable at the crime scene [19].

### The layouts

**Fig. 1:**
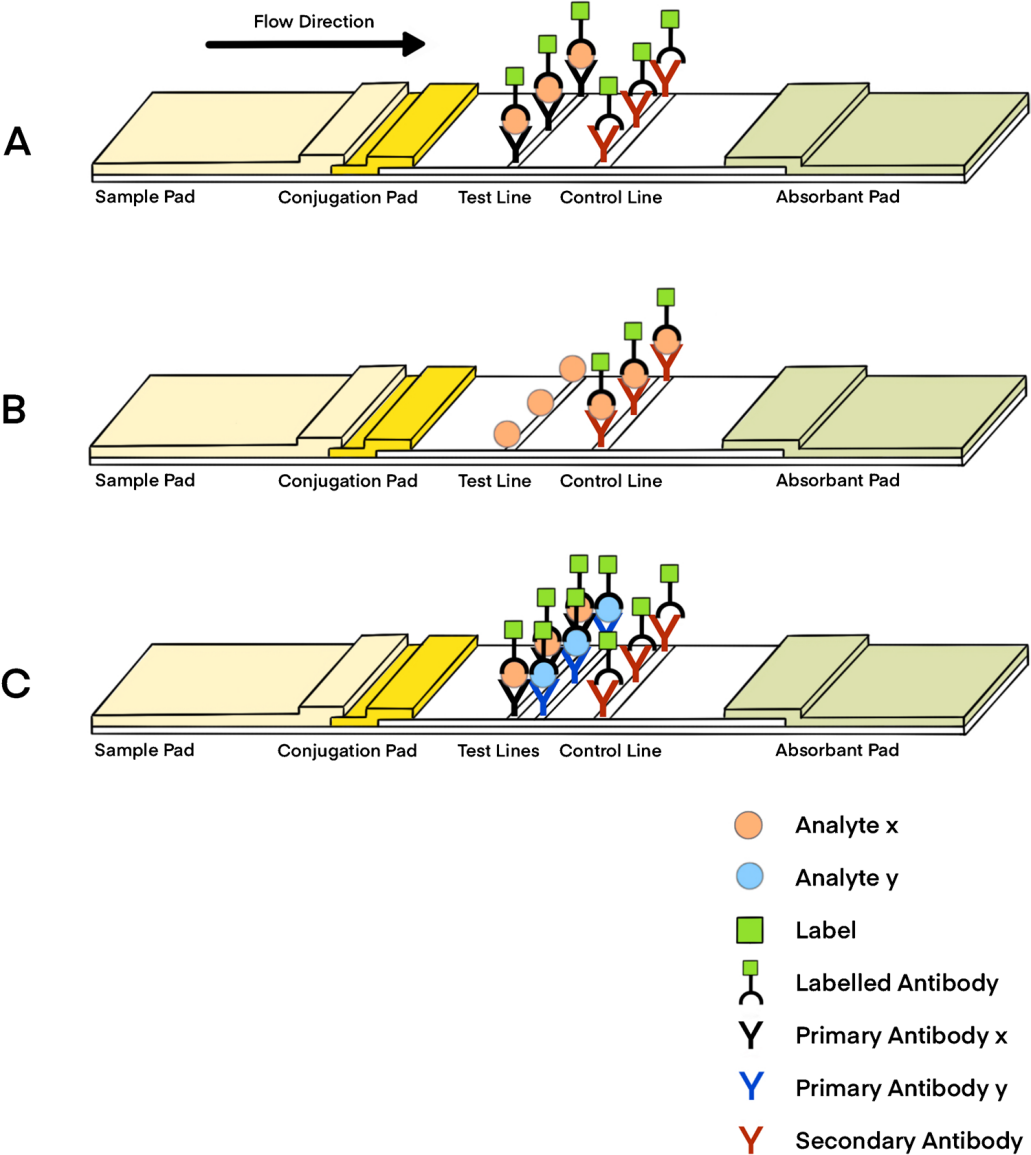
Different LFA layouts. **A** Noncompetitive layout: if present, the analyte in the sample is captured on the test line between the primary antibody *x* and the labelled antibody. The labelled antibody will always attach to the secondary antibody on the control line. **B** Competitive layout: if present, the analyte in the sample will compete with the analyte on the test line for binding to the labelled antibody. Some labelled antibodies may attach to the analytes on the test line. The signal intensity on the test line is inversely proportional to the amount of analyte in the sample. Thus, the appearance of only the control line indicates a strong positive result. **C** Multiplex layout: two distinct analyte molecules, *x* and *y*, can be detected on the same strip by immobilizing specific primary antibodies, *x* and *y*, respectively, on the test lines

The versatility of the LFA technique is in part attributed to the availability of various layouts. The selection of a particular layout depends on the type and purpose of the analysis. One prevalent layout is the noncompetitive assay. This layout is the most widely implemented in forensic applications (e.g., tests from Independent Forensics, SERATEC, and BlueSTAR-forensics) due to its high specificity and the ability to accurately and precisely identify the analyte [14, 20]. In this format, the capture antibody specific to the analyte is immobilized on the test line of the strip while a secondary antibody targeting the detection antibody that is conjugated to the visual enhancer is immobilized on the control line (Fig. 1A) [21]. The colored control line affirms the proper functionality of assay and confirms the suitability of the utilized antibodies for the test. The colored test line will signal the presence of the analyte in the sample and the color intensity will be proportional to the amount of that analyte present in the analyzed sample volume. In this manner, the LFA can provide outcomes that are both qualitative and semi-quantitative [19]. Depending on the type of visual enhancer that is implemented, a flatbed scanner or digital camera can provide a more accurate quantification of the emitted signal [22]. Depending on the available scanner, this is a cheap and easy-to-use technique that yields results that are comparable to the ones acquired using expensive laboratory equipment and immunoassay experiments. A noteworthy limitation of LFA assays lies in their reduced specificity, wherein challenges arising from potential cross-reactivity can significantly impact the quality and accuracy of the result [23, 24]. In the noncompetitive layout, the incorporation of two distinct antibodies reduces the likelihood of non-specific interactions, distinguishing it from alternative immunoassay layouts and thus enhancing specificity [20].

Another layout frequently encountered for the detection of small analytes in the sample is the competitive immunoassay [23]. In contrast to the noncompetitive layout, the appearance of two-colored lines on the strip, that of the test and the control, will indicate the absence of the analyte in the sample (Fig. 1B) [19, 25]. Similar to the noncompetitive assay, this layout allows for a qualitative and semi-quantitative analysis [26]. The interpretation of the signal emitted upon interaction depends on the type of visual enhancer implemented in the assay. Historically, the competitive immunoassays implemented radioactive enhancers and as a result less signal would be obtained at higher analyte concentrations [27]. Competitive immunoassays can facilitate a qualitative analysis by naked eye since low analyte concentrations may be detected by emission of an intense signal [20]. However, they may be more susceptible to interference effects (e.g., sample purity, running/washing buffers, and antibodies) with respect to the noncompetitive assays since only one capture antibody is used [24, 28].

Lastly, the multiplex detection layout is a recently advanced approach that aims to investigate an array of relevant analytes on the same strip and under the same conditions (Fig. 1C) [19]. The assay is characterized by high versatility since the size and shape of the strip can be modelled to accommodate multiple test lines. The amount of acquired information greatly increases while potential inter-test variability is reduced. Nevertheless, issues concerning the assay’s specificity and sensitivity may become more prominent. Given the recent practical implementation in disease epidemiology of this layout, further insights are imperative before formulating any definitive conclusions [19, 29, 30].

## Conjugation techniques

The success of the LFA test lies on the visual and rapid inspection of the results. The LFA aims to overcome the limitations posed by more intricate and time-intensive analytical methods while maintaining a high accuracy and sensitivity [31]. The previous section highlighted the different LFA layouts and their strengths for compound detection. In addition, the type of visual enhancer implemented in the assay will greatly impact the overall efficiency of the assay. A successful interaction needs to be signaled using adequate visual enhancers that are specifically conjugated to the detection antibody while preserving the binding efficiency of the latter to the analyte and other compounds [24]. Visual enhancers that are implemented in the LFA for forensic purposes must be versatile and remain stable at different conditions and uncontrolled temperatures if analysis is carried out at the crime scene. Moreover, the enhancers must present a reproducible conjugation efficiency through a straightforward approach, to maintain the simplistic character of the LFA. Alternatively, if in-house production of the enhancers proves unfeasible, it is imperative that these are commercially available and at a relatively low cost [32].

### Gold nanoparticles (AuNPs)

These are the most frequently implemented visual enhancers in the LFA due to their facile binding capacity with other molecules and their versatility [19]. AuNPs are spherical particles that can vary in size upon chemical manipulation [33]. More precisely, varying the production conditions (e.g., amount of dopant, oxidation state) will allow to tailor the size of the AuNPs [34]. Both the size and shape of the particles will determine the resulting optical properties and stability of the enhancer [33]. Usually, the diameter of the AuNPs that are implemented ranges from 4 to 323 nanometers (nm) [35–37]. Moreover, these colloidal gold enhancers are characterized by simple preparation and scalable production with minimized environmental impact. They demonstrate stability across varying conditions and possess a high affinity for biomolecules [19, 36]. A detailed protocol for in situ production has been previously described by Lin et al. [36]. The conjugation of the AuNPs to the antibodies or other molecules may be achieved by establishing (non)covalent interactions (e.g., van der Waals force, hydrophobic interactions). A successful conjugation can be monitored by an increase in the absorbance peak of the AuNPs [33]. The most frequently encountered method for detecting the conjugated product, AuNPs-antibodies, and observing the corresponding spectra is the X-ray photoelectron spectroscopy (XPS) [36]. The amount of the AuNP enhancers used in the LFA will be proportional to the intensity of the emitted signal on the test line (Fig. 5a) [33]. The implementation of AuNPs as enhancers in a LFA enables (semi-)quantitative analysis by visual inspection or the use of optical strip readers [19]. The AuNPs are characterized by high accuracy (error rate less than 10%), low cross-reactivity, and specificity [37, 38]. Such characteristics enable AuNPs to be extensively utilized as visual enhancers in the LFA setup for the field of clinical diagnostics (e.g., cancer biomarkers, osteoprotegerin, CEA-tumor marker) [36, 39, 40]. The AuNPs allow for a sensitive analysis with low detection thresholds that range in the order of picograms/mL-femtograms/mL [36, 38, 40]. Furthermore, to guarantee the accuracy and reproducibility of the LFA test, stabilization agents (i.e., bovine serum albumin, polymers, organic cations) for the AuNPs-antibody conjugate can be used. Their implementation will minimize the impact of matrix effects and increase the test’s specificity, thus serving as blocking agents [33]. The efficacy of AuNPs as visual enhancers in LFAs is attributed to their facile functionalization, enabling an augmentation of the signal emitted on the strip lines and consequently enhancing the method’s sensitivity [19]. Diverse strategies can be employed to achieve this enhancement. One such approach involves the utilization of immunogold silver staining. The silver ions will create a film around the AuNPs that may result to a 100-fold enhancement of the assay’s sensitivity [41]. However, such manipulation would increase the costs, time of the total reaction, and complexity of the assay. An alternative method for signal enhancement entails the use of the AuNPs as carriers for specific enzymes. For instance, in a noncompetitive layout, such functionalization can be practically achieved by bounding the antibody, that will be conjugated to the enhancer, to a specific enzyme (e.g., HRP). The secondary antibody that is immobilized on the strip will carry the substrate for the specific enzyme (e.g., 3,3′,5,5′-tetramethylbenzidine, 3,3'-diaminobenzidine). The AuNPs that are conjugated to the modified antibody bearing the enzyme (e.g., HRP) will carry the complex to the immobilized secondary antibody and upon successful interaction a darker and more intense color will be emitted on the test strip line, resulting in better sensitivity and quality of the results [37]. Overall, the enzymatic enhancement of the AuNPs can improve the sensitivity and lower the detection threshold (pg/mL) [35]. Various commercially available enzymes, such as alkaline phosphatase and β-galactosidase, exhibit the capability to generate a robust signal upon interaction with a designated substrate. This attribute enhances the versatility of the technique [42]. However, it is crucial to select the appropriate substrate to ensure maximum enzyme–substrate affinity, thereby achieving the highest sensitivity [19, 43, 44]. Among the limitations associated with the enzymatic signal enhancement of AuNPs, susceptibility to experimental and/or environmental conditions is observed, rendering them more prone to degradation [45]. Furthermore, the incorporation of enzymes substantially increases both the overall costs and analysis time due to the intricate preparation and purification steps involved [36, 45].

### Magnetic nanoparticles (MNPs)

**Fig. 2:**
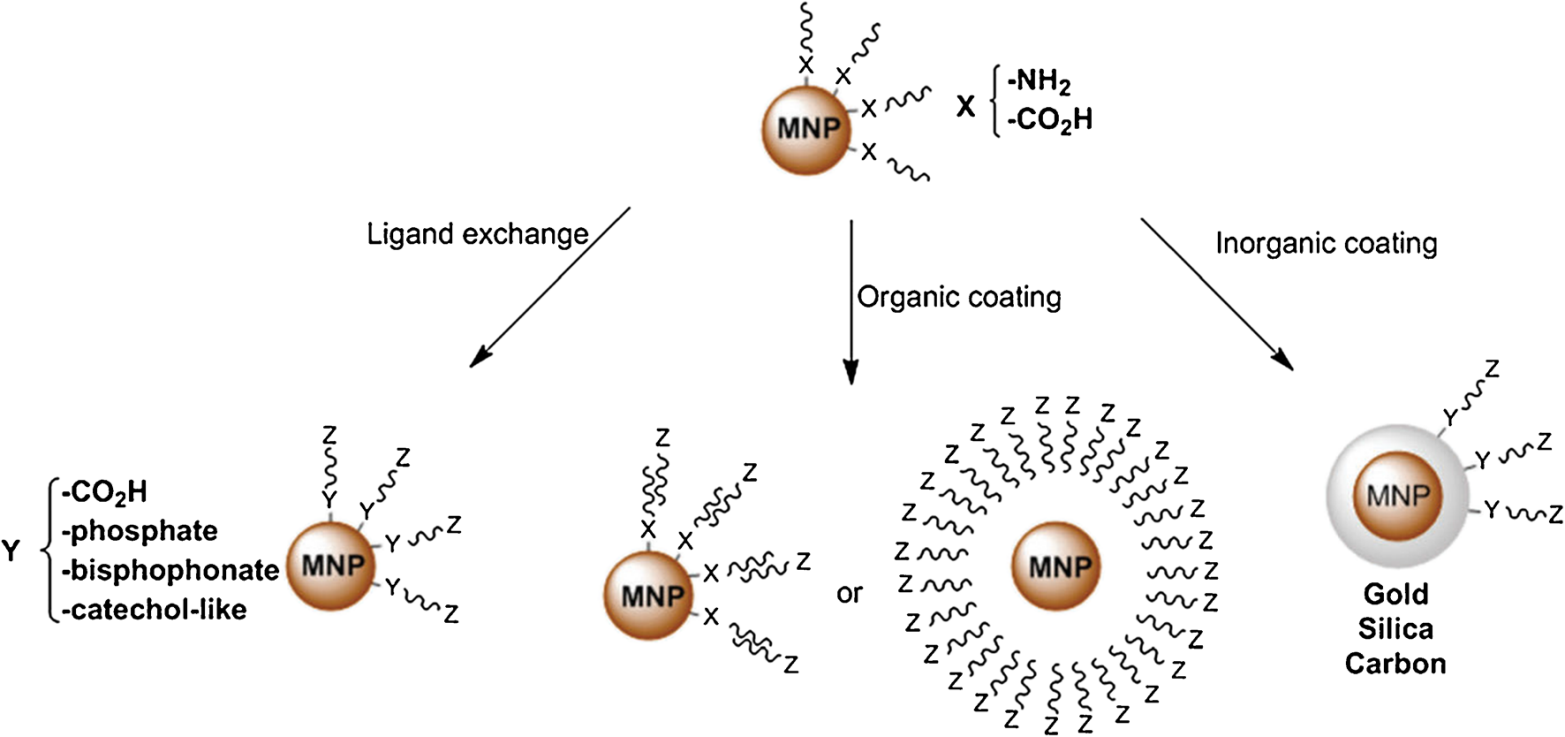
Overview of the different functionalization methods of MNPs for subsequent conjugation [51]

The use of MNPs as visual enhancers in the LFA setup shows great potential for a rapid (semi-)quantitative analysis. Recent advances in the field of clinical diagnostics can support such assertion [19, 46]. These beads are characterized by increased stability over time and can increase the LFA’s sensitivity up to 100-folds. The detection limit of MNPs is approximately 0.25 ng/mL [47]. Since magnetic interferents can be rarely found in the surrounding environment or in the sample volume, a low background noise can be expected and thus, a higher contrast in the obtained strips [33]. The MNPs consist of a core (e.g., Fe_3_O_4_, nickel, neodymium, magnetite) coated with adequate chemical molecules (e.g., amine, carboxyl, epoxy, tosyl) that conjugate to the antibodies and form the immunomagnetic beads [33, 48, 49]. The functionalized beads are commercially available; however, protocols are in place for further optimization coating [48, 50]. Prevalently, functionalized MNPs with a Fe_3_O_4_ core and a silica or chitosan coating are employed to guarantee covalent binding and increased biocompatibility [50, 51]. The silica coating will guarantee the presence of amine groups that bind in a stable manner the carboxy groups of the targeted antibodies. The chitosan coating will also ensure stable interactions. However, the chitosan’s polymer chains will increase the overall size of the magnetic particles (Fig. 2) [51]. A successful functionalization of the MNPs can be confirmed by Fourier transform (FTIR) and/or transmission electron microscopy (TEM) [50].

The MNPs will produce a dark brown color upon successful interaction and aggregation on the test line (Fig. 5c). The immunomagnetic beads bound to the analyte can maintain their absorption properties, allowing the end user to obtain accurate results long after the assay’s completion [49, 52]. The intensity of such color can be observed to provide an indication of the analyte’s abundance in the sample for a qualitative assessment [19]. However, with respect to the AuNPs, the resulting color will not be as intense [49]. For this reason, the unique ability of the MNPs to emit magnetic signals upon interaction can be used for the quantification of the analyte’s concentration in the sample. For such quantification and enhancement of the method’s sensitivity, a magnetic reader instrument must be coupled to the LFA [19]. These MNPs are vastly implemented as visual enhancers in LFA tests due to their versatility. The size and nature of the magnet present in the core of these particles will influence the detection threshold and the duration of the assay [52, 53]. The size of the MNPs spans from 1 to 200 nm. Generally, the size exerts an influence on the flow rate and thus, the detection time [52, 54, 55]. The ideal MNPs must have the smallest size and the highest magnetite content that is uniformly distributed for a fast and sensitive detection [42]. Therefore, an adequate MNP that is fit-for-purpose will further allow the refinement of the sensitivity. Commonly, since MNPs can remain stable, smaller particles are preferred which will confer greater magnetic power and velocity [53]. Moreover, commercially available MNPs exist that can be further modified and optimized prior to their implementation [55]. A novel visual enhancer, the gold magnetic bifunctional nanobeads (GMBN), was introduced that combines the strengths of both AuNPs and MNPs to increase sensitivity and efficiency of the LFA [56]. The Au core allows for a noncovalent conjugation to the antibodies, while the Fe_3_O_4_ shell separates and minimizes matrix effects arising in the presence of a magnetic field [33]. Another promising visual enhancer combines the core of MNPs and a dual quantum dot shell to increase the sensitivity of the LFA by merging the advantageous properties of both enhancers [57]. While MNPs show great conjugation and storage stability, easy functionalization, and low background noise, there are limitations that should be considered. One noteworthy consideration is the vulnerability of the MNPs to external magnetic forces, which may exert influence on their positioning and functionality [58].

### Fluorescent/luminescent material

Different types of fluorescent enhancers are extensively used in the LFA. Upon successful interaction on the strip lines, the excitation and emission of a fluorescent signal can be used for quantification purposes. Furthermore, a more accurate quantification of the signal can be achieved by coupling the LFA to a portable fluorescent strip reader [19]. One of the most important advantages of the fluorescent enhancers is the absence of a background signal; thus, they allow for a more sensitive quantification of the results [18]. However, fluorescent enhancers require prior excitation through the implementation of light sources tailored to their optimal excitation wavelength spectra. Depending on the type of enhancer, this process might degrade the samples and impede subsequent STR-DNA laboratory analysis [59]. Moreover, fluorescent enhancers are susceptible to photobleaching, a phenomenon of chemical and/or metabolic degradation. Photobleaching might have an effect on the stability of the fluorescent enhancer and therefore, on the detection efficiency of the analyte [19, 60]. Finally, the functionalization of fluorescent enhancers and the preparation steps are elaborate and can result in inter-batch variability that eventually will impact the reproducibility of the assay [61]. Among the commonly implemented fluorescent enhancers for the detection of analytes in the sample are the organic fluorophore dyes. These dyes are characterized by higher sensitivity with respect to the AuNPs (up to 100-fold higher) [19, 61]. The size of the dyes ranges between 1 and 10 nm [62]. The fluorescent molecules are protected inside a silica matrix, thus conferring increased stability. Moreover, they can be easily functionalized since the surface of the matrix can be modified to present specific reactive sites. For instance, the cyanine5 is a dye that emits in the red spectral area. This dye can be encapsulated inside the silica matrix while maintaining its initial behavior and properties. The characteristic red emission spectra will allow for a clear distinction from non-specific fluorescent signals from the sample or background noise and will thus minimize the risk of false positive results [61, 63]. Other dyes that can be implemented as enhancers for achieving low limits of detection are the near-infrared fluorescent dyes [64].

**Fig. 3:**
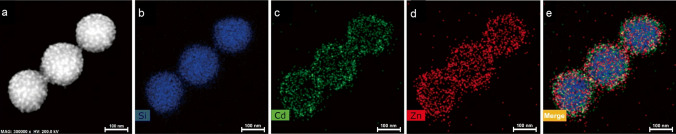
**a**–**e** Elemental mapping results of a SiO_2_@QD nanoparticle (image adapted) [72]

Another type of fluorescent enhancers are the quantum dots (QDs), semiconducting particles with unique optical properties that are highly versatile. They have been implemented in the clinical framework for targeting biomarkers in a specific manner [65]. The core of the QDs can constitute of cadmium selenide (CdSe), cadmium telluride (CdTe), indium phosphide (InP), or zinc selenide (ZnSe). The core is encapsulated by the protective shell, silica, or ZnSe (Fig. 3) [66, 67]. The functionalization of the QDs can be achieved by solubilizing and thus, exposing the carboxylic groups of the shell for subsequent covalent bonding [68]. Depending on the type of shell, there will be carboxy- or amine- groups forming stable and oriented bonds with the Fc regions of the antibodies [65]. Various protocols are available for the production of stable QD enhancers and guidelines for their functionalization [67]. A correct synthesis of the enhancer can be further validated by TEM [69]. The characteristics of the QDs can be easily modelled and depend on their size and shape [19]. The size of the QDs spans from 3 to 100 nm [68–70]. By tuning their size, the emission spectra will change, from blue to near infrared light. There are commercially available QDs of different sizes and coatings [71]. They can be used in a multiplex detection format where the QDs are functionalized to have distinct narrow emission peaks upon excitation with a single light source [33, 72, 73].

The results can be observed almost immediately when the LFA is coupled to a portable strip reader that will measure the fluorescence signal emitted upon successful interaction of the conjugated antibody with the analyte in the sample (Fig. 5d). The intensity of the fluorescent signal will be directly proportional to the amount of analyte present in the sample [19]. However, the presence of a strip reader is not essential for the detection of low analyte concentrations and the semi-quantification. For instance, a concentration as low as 10 ng/mL of human 13 ceruloplasmin can be detected by visual inspection of the developed color on the strip [19]. Overall, the limit of detection is in the order of picograms/mL [70]. Moreover, QDs are characterized by high stability and resistance to photobleaching [19, 73]. They are thought to be 100 times more stable than conventional organic dyes [68]. The ability of the QDs to resist extreme conditions (e.g., heat, chemical oxidation) is conferred by the outer protective shell and the polymer cross-link modifications [74]. Therefore, a thick shell layer will provide chemical stability. However, an increase in the thickness will negatively affect the quantum efficiency and thus sensitivity. Optimization steps such as tailoring the shell’s thickness or adding an additional alloy shell between the core and the outer shell of the QDs are essential prior to implementation in the LFA [67]. Moreover, QDs have high absorption coefficients and brightness, both necessary for a sensitive analysis [19, 33]. However, drawbacks of such enhancers should not be disregarded. QDs are difficult to shape and to functionalize, and toxicity/sustainability issues may arise during the production and implementation phases. The latter is mainly due to the presence of metal ions (e.g., cadmium) inside the core of these enhancers [69, 75]. Therefore, their application is limited and restricted to operators with a certain level of expertise [19]. In addition, there are risks of non-specific binding of QDs on the test line when the analyte is absent. However, the risk of such event can be minimized by implementing adequate blocking steps of the nitrocellulose membrane (e.g., bovine serum albumin, casein) [73].

The luminescent lanthanide nanoparticles may be used as visual enhancers in the LFA test. They are characterized by high sensitivity since they minimize the background fluorescence interference [33]. The size diameter of these nanoparticles is approximately 20 nm [76]. Owing to their versatile nature, the sensitivity of the assay can be fine-tuned by modulating the size of these particles in accordance with the analyte type and increasing their affinity during the functionalization stage [18]. The limit of detection of the lanthanide nanoparticles ranges between 0.01 and 0.058 ng/mL [77]. When compared to the AuNPs, the lanthanide particles present a lower detection limit (approximately 100-fold more sensitive). Upon successful interaction, they produce a characteristic red coloring on the strip rendering the result interpretation in real-time and in situ semi-quantification of the bands’ signal feasible. A distinctive attribute of these enhancers lies in their capacity for result visualization through direct inspection with the naked eye, eliminating the requirement for an additional strip reader. Simultaneously, they maintain stability under diverse environmental conditions. However, for the visual inspection of the results, it is important to consider the subjective nature of the observations [18, 77]. Depending on the observations of the end user and/or the sample composition different conclusions will be reached causing reproducibility concerns. Moreover, despite the stability of the lanthanide particles, they may cross-react with other components in the sample volume [19].

A similar type of fluorescent enhancer that is used in the LFA are the upconverting phosphors (UCP). These enhancers contain lanthanide chelate particles in their core [33]. Their unique optical feature allows them to emit light in the visible range upon excitation in the infrared red region (Fig. 5e) [19, 33]. They are characterized by increased stability, sensitivity, and low-cost production [33]. Furthermore, the coupling of the LFA strips to a portable reader or smartphone device can render the acquisition of quantitative results at the crime scene, feasible, rapid, and inexpensive. The reader can measure the fluorescent signal on the strip lines and the background noise to quantify the analyte concentration in the sample in an objective manner that will be less operator-dependent. Therefore, such reader will contribute to the reproducibility of the results and commercialization of the method [78].

Alternative fluorescent enhancers implemented in the LFA for a rapid and sensitive analysis are the fluorescent microspheres (FMs) [79, 80]. The FMs are stable over time and can yield a high fluorescence signal, thus increasing the assay’s sensitivity [33, 80]. The FMs can be multicolored and upon excitation, the fluorescent signal will appear as a narrow peak that will allow a detailed discrimination. Similarly, the LFA test can be coupled to a portable fluorescence reader for a rapid detection, in approximately 10 min, and quantification of the response signal [79]. However, one important drawback of the FMs is that their affinity and the velocity of the conjugation is greatly depended on the concentration of targeted antibodies. The relation between those two will be proportional; an augmentation in the antibody concentration will result in an accelerated conjugation to the enhancer [33]. Moreover, due to the high sensitivity conferred by the FMs, impurities in the sample will further impact on the detection efficiency. Thus, this method requires a prior purification of the sample to optimize the results and guarantee reproducibility. Such pre-treatment may not be possible in forensics since the volume of the available samples is limited and a purification step could irreversibly degrade the trace evidence. Moreover, an elaborate sample preparation would require equipped laboratory and trained personnel, thus increasing the overall operational costs [79].

### Latex beads

These colored labels are implemented in the LFA as a less expensive alternative to the AuNPs. The latex beads are pre-treated with common dyes (e.g., blue, black, or red) and used for a rapid and easy detection of the analyte in the sample [81]. The size of these beads ranges from 100 to 1000 nm, and they are commercially available [82]. The latex beads can be either coated directly with the target antibody or with streptavidin. In the latter scenario, the functional group of streptavidin will cross-link with the biotinylated target antibody. Diverse commercial protocols are available to produce such conjugates [83]. The successful interaction with the investigated analyte will be signaled by a colored test line on the strip. The identified limit of detection of these beads is 1.0 ng/mL [84]. The use of different colors can allow a multiple analytes analysis that would facilitate a rapid screening of trace elements found at the crime scene. Furthermore, the conjugated latex beads can be stable from 2 months to 2 years. The latter applies for the antibody-coated beads [83]. However, latex beads are characterized by lower sensitivity, compared to AuNPs or QDs, and the interpretation of the results mainly depends on the operator’s visual inspection [70, 84]. Additionally, in absence of or low antibody quantity, the beads tend to aggregate due to van der Waals’ forces and thus, colloidal stability issues become more prominent. Addressing such issues necessitates the incorporation of specific counteracting additive [85].

### Colloidal carbon/carbon nanoparticles (CNPs)

**Fig. 4:**
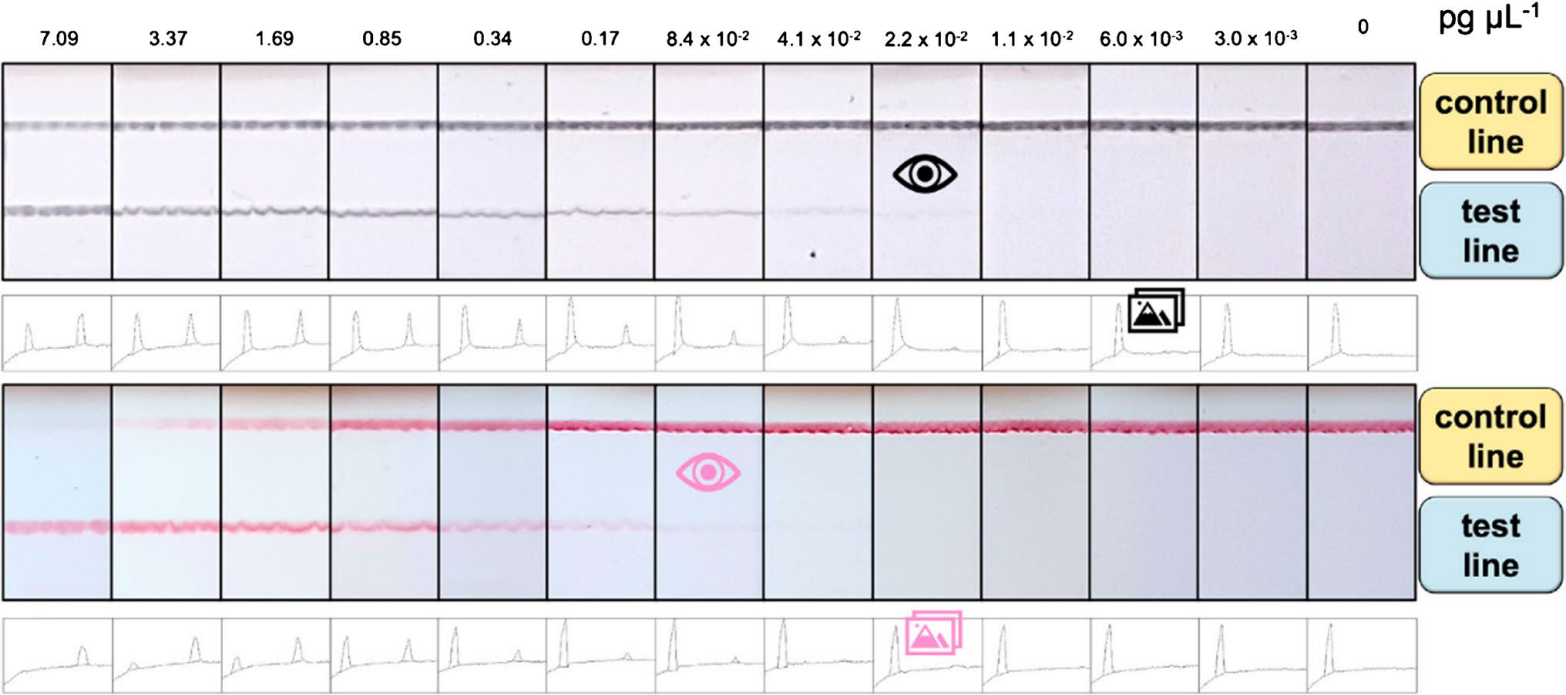
Detection of *E*. *coli*, direct comparison of the limit of detection by visual inspection for functionalized CNPs (upper panel) and functionalized AuNPs (lower panel) [92]. From left to right, decreasing analyte concentrations are examined. With Spezial Schwarz 4 CNPs, a signal on the test line can be observed down to 2.2 × 10⁻^2^ pg/μL of analyte. With AuNPs, no signal can be observed for analyte concentrations below 8.4 × 10⁻^2^ pg/μL

The implementation of CNPs as visual enhancers in the LFA setup has not been extensively documented [31]. However, existing literature has reported successful applications of CNPs (e.g., Special Black 4, Vivacta, Maiia) in the fields of food and agriculture [31, 86]. Consequently, the advantageous properties exhibited by these enhancers can be proven beneficial in the context of forensic analysis. The size of the CNPs spans from 25 to 250 nm [87]. Commercially available CNPs such as SB4 with a 120-nm diameter, are characterized by high costs [88]. Conversely, a variety of protocols for the synthesis of the CNPs starting from low-cost material are available [86, 89–91]. The CNPs can be coated with streptavidin or neutravidin while the target antibodies can be tagged with biotin and digoxigenin respectively for further signal enhancement. The conjugation of the CNPs to the antibodies can be achieved through (non)covalent interactions, and the validation can be achieved through TEM [88–90, 92]. Overall, the detection threshold of CNPs is comparable to that of the enzyme-linked immunosorbent assay (ELISA), a more sophisticated technique utilized for compound detection [19]. CNPs exhibit enhanced sensitivity compared to the previously examined nanoparticles [93]. Specifically, the limit of detection for the Spezial Schwarz 4 (Degussa AG, Frankfurt, Germany) CNPs is 2.2 × 10⁻^2^ pg/μL, which is lower than that of AuNPs at 8.4 × 10⁻^2^ pg/μL (Fig. 4) [92]. Additionally, amorphous CNPs have been shown to possess sensitivity that is twofold to eightfold greater than that of latex beads and QDs, respectively [88].

**Fig. 5:**
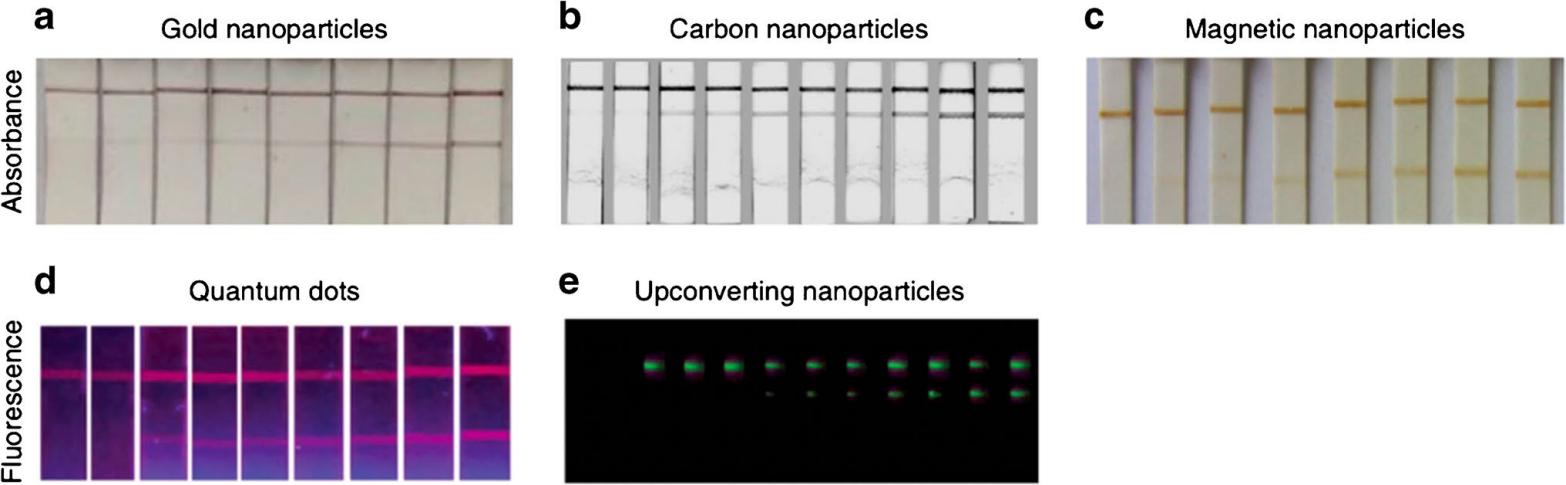
Overview of different strip readouts when specific visual enhancers are implemented (image adapted) [95]

Furthermore, CNPs are stable and thus suitable for an accurate analysis [86]. In a study by Zhang et al. the precision of CNPs, obtained from candle soots, was assessed. Over a duration of 35 days following conjugation, the enhancers exhibited sustained precision. The observed coefficient of variation within this period ranged from 9 to 13% [88]. Other studies investigating the Spezial Schwarz 4 (Degussa AG, Frankfurt, Germany) CNPs’ stability showed how they remain conjugated for longer periods (i.e., wet conjugates for 1 year at 4 °C and the dry conjugates for 3 months at room temperature) [87]. Characteristics of these CNPs such as low-cost and simplistic production, straightforward conjugation procedures, and their prolonged stability make them optimal candidates for the visualization and enhancement of the results on the strip [31]. Upon a successful interaction of the antibody with the analyte, the resulting black color on the strip provides a high contrast that can easily be observed even for lower analyte concentrations in the sample volume (Fig. 5b) [19, 33]. The Spezial Schwarz 4 CNPs are versatile and can be used for visually enhancing different compounds [19]. Issues such as cross-reactivity and background signal noise can be addressed by utilizing different antibody tags to establish more specific and robust conjugation to the CNPs [91]. The Spezial Schwarz 4 CNPs can bind to compounds by direct absorption and will remain stable in suspension without the use of any stabilizing agents. Furthermore, the risk of nonspecific binding to protein and/or DNA molecules in suspension can be minimized by reducing the number of available functional groups on the carbon particle’s surface [31]. Through this simple preparation and functionalization process, the CNPs can be oriented to bind to the target molecules with high affinity. Further signal enhancement can be achieved by coupling the CNPs to enzymes such as HRP-red [94]. In particular, the neutravidin coating can be fused with the HRP enzyme; such enhancers are commercially available [91]. Finally, the CNPs have an amorphous nature and heterogeneous particle size. Thus, the smaller particles will migrate faster, bind to the analyte, and allow for a rapid detection [31]. However, CNPs present some limitations mainly due to their limited implementation and restricted commercial availability [31, 88, 94]. The in-house production and functionalization of Spezial Schwarz 4 CNPs may give rise to intra-test variability and lack of reproducibility [94]. Upon production, the CNPs tend to form aggregates (> 200 nm) that require monitoring and frequent sonication prior to use [92]. Additionally, the functionalization of the amorphous particles through non-specific protein adsorption can impact on the specificity and reproducibility of the LFA tests [92, 94]. One aspect to be considered is the constraint in utility rising from the inherent black coloration produced upon interaction [31]. As previously noted, a proposed solution to augment or modify the signal color involves the coupling of CNPs to enzymes. However, this approach introduces an increased risk of false positive outcomes in the absence of the analyte, attributable to the background strip coloring [94]. Additionally, the development of luminescent CNPs would increase the overall costs and necessitate the use of a dedicated reader [31].

## Discussion

The purpose of the present work is to identify suitable visual enhancers that can be used in a LFA setup for a sensitive and accurate forensic trace analysis. Through an extensive literature search on past and current developments, different enhancers are investigated and critically compared. The criteria selected for evaluating the efficiency of an enhancer in a LFA setup included sensitivity, reproducibility, visualization, costs, size, enhancement potential, and type of signal. An efficient visual enhancer must adhere to these stringent criteria to be deemed suitable for implementation in forensic practices. However, each investigation presents unique circumstances. In instances of high-volume crimes, a cost-effective enhancer may be preferred, whereas investigations involving challenging samples may necessitate a more expensive enhancer (e.g., fluorescent QDs, AuNPs) to enhance the assay’s sensitivity. This indicates that certain criteria may hold more significance depending on the specific case. Nevertheless, sensitivity and reproducibility criteria must be fulfilled in every case. The use of a noncompetitive LFA coupled with CNPs as visual enhancers is considered to be an efficient setup that complies with the high forensic standards.

Since novel technological developments constantly arise, the world of forensics puts forward appointed committees and strict guidelines for an adequate implementation of the methods [96]. The extensive number of biological traces present at crime scenes poses a substantial challenge to the analysis procedure, resulting in prolonged processing times and elevated costs [1, 2, 97]. The laborious forensic practices that are currently in place have been criticized for the lack of accuracy, reliability, and transparency [98]. To overcome these limitations and advance the field of forensics, there is an increasing interest to develop scientifically grounded and robust methodologies that enable the chemical profiling of biological traces [16, 99, 100]. In reviewing the literature, immunolabelling, gas chromatography coupled to mass spectrometry (GC–MS), and other sophisticated techniques (e.g., MS/MS, LC–MS) are used for the detection of chemical compounds [100–102]. Despite the success and accuracy of these analytical methods in obtaining the chemical profile of the examined traces, their implementation in routine case work is limited. The need for dedicated workspace, expensive instrumentation, and trained personnel greatly increases the overall cost of the investigation [103, 104]. LFA can be a promising alternative as a one-step analysis for the extraction of valuable information [19]. Commercial LFA tests are available for presumptive testing including the different RSID tests for body fluid identification [14, 105]. However, further chemical profile analysis is proposed for complex cases where there is insufficient DNA quantity and quality for STR profiling and/or the fingermarks would normally be disregarded since they do not reach the quality thresholds for the traditional approaches [106]. For this, adherence to the established criteria for determining the efficiency is crucial. Furthermore, the LFA is a fast (approximately 10–20 min) and user-friendly analytical method. Lastly, it is portable, miniaturized, and inexpensive [107]. The noncompetitive immunoassay is characterized by increased specificity [20]. It implements two antibodies (i.e., the capture and the detection) that are specific for the analyte and thus minimize the risks of cross-reactivity that would lead to false positive/negative results. Therefore, for implementation in forensic casework, it is the preferred layout over a competitive or a multiplex assay (Fig. 1).

The present study thoroughly examines the most commonly implemented enhancers in the LFA and critically reflects on their strengths and limitations. An overview can be found in Table 1. More precisely, the AuNPs are small sized enhancers, capable of fast migration and proper flow rate on the strip [35–37]. Their low detection limits (~ pg/mL) and strong emission signals render them suitable for the analysis of crime scene traces [38, 40, 41]. For challenging samples such as degraded or mixed-profile stains, the AuNPs’ signal can be further enhanced to detect analytes present in lower amounts [37]. The characteristic red signal can be visually inspected and/or when coupled to a reader can be recorded. The intensity of the signal can be compared to a standard calibration curve and semi-quantitative information can be extrapolated. However, commercial AuNPs are expensive for routine casework implementation [36, 45]. In-house production of AuNPs can exhibit batch-to-batch variation and thus, repeatability issues. Available solutions for enhancing the stability of the AuNPs (e.g., BSA, polymers, organic cations) will increase the production complexity and costs. Lack of reproducibility and low throughput in the production phase are generally not preferred for forensic analysis. The MNPs allow for good flow rate owing to their small dimensions [52, 54, 55]. They produce a dark brown color that is less intense than that of the AuNPs, but that still allows for visual inspection of the results [49]. The MNPs are stable with a LOD of 0.25 ng/mL. However, in the presence of external magnetic forces, their performance is greatly impaired, which causes concerns over the assay’s robustness [58]. Overall, MNPs are cheaper than AuNPs and QDs. Nevertheless, cheaper alternatives are available with similar LODs. The organic fluorophores have extremely small sizes that have the potential to increase the sensitivity of the assay. Indeed, they are 100-fold more sensitive than the AuNPs and are relatively cheap [61]. However, the organic fluorophores are extremely unstable. During crime scene investigations it is difficult to control the environmental conditions (e.g., light sources, temperature, humidity). Issues such as photobleaching of the fluorophores can have a severe impact on the analysis and the interpretation of the results [60]. Forensic practice has little tolerance for intra-assay variability, both sensitivity and reproducibility criteria must be fulfilled. Furthermore, the organic fluorophores require coupling to a UV or fluorescence-reader for the visualization of the results. The implementation of a reader will increase the overall costs of the investigation even though the initial costs for acquiring the enhancers are low. The fluorescent QDs range between 3 and 100 nm in size, with low detection limits, and high stability [68–70]. These fluorescent enhancers overcome the abovementioned challenges of the MNPs and organic fluorophores. Nonetheless, and similarly to AuNPs, due to their relatively high costs, often they are not the preferred visual enhancer for forensic routine application, high-volume crimes. Furthermore, the presence of cadmium or other metal ions in their core poses significant toxicity concerns for personnel, specifically crime scene examiners [69]. In recent years, bodies of research focus their efforts on the development of green synthesis routes. Plant extracts (e.g., Mexican mint, *Cucumis melo*) as precursors for the QDs synthesis promise to be more sustainable and cost-effective alternatives [75]. The lanthanide nanoparticles are extremely small in size with very low detection limits [77]. However, issues of inter-assay variability and cross-reactivity are prominent in lanthanide enhancers. For forensic applications, it is imperative that both sensitivity and reproducibility criteria are fulfilled. The toxicity and accuracy issues in conjunction to their cost render them a less suitable enhancer candidate for the LFA. Latex beads have a bigger size that will impact the sensitivity of the assay. Their detection limit is higher than that of the other visual enhancers [70, 84]. Nevertheless, they are largely implemented as a less expensive alternative to AuNPs [81]. A notable benefit of utilizing latex beads resides in their capacity to employ different colored dyes for multiplex analysis. In the multiplex format, less sample is required and the overall processing is accelerated. However, in the field of forensics a reduced sensitivity is not preferred since this increases the risk of false negative results and therefore can impact the entire investigation. Finally, the CNPs (e.g., Spezial Schwarz 4) are small sized enhancers of extremely high sensitivity [19, 87, 88, 92]. In the presence of the target analyte, they produce a characteristic black color that can be easily visualized due to the pronounced contrast against the nitrocellulose white background membrane. They are affordable and extremely stable visual enhancers. They can be easily produced in-house, thus minimizing the risks that are associated with a possible discontinuation of commercial products and its impact on the standard forensic procedures. Their high stability also reduces the risks of batch-to-batch variation and thus, allows for a reproducible analysis [88, 92]. Moreover, the CNPs can be easily functionalized with enzymes such as HRP for a dual color visualization and increased sensitivity. However, any additional signal enhancement effort will increase the overall production costs. The CNPs can also, be used in a multiplex format by conjugating them to different target antibodies, and thus, exhibiting increased versatility. However, in the forensic field, even minimal batch-to-batch variation in CNPs’ performance cannot be overlooked, particularly when analyzing trace amounts of analytes. Carbon-based enhancers are more prone to aggregation and sedimentation, especially in colloidal suspensions. Companies such as Genzyme Diagnostics, Vivacta, and Maiia have made efforts to patent and commercialize CNPs [31]. Nevertheless, the absence of standardized production processes and the complexities surrounding regulation and legislation limit the applicability of CNPs for forensic purposes.

**Table 1:**
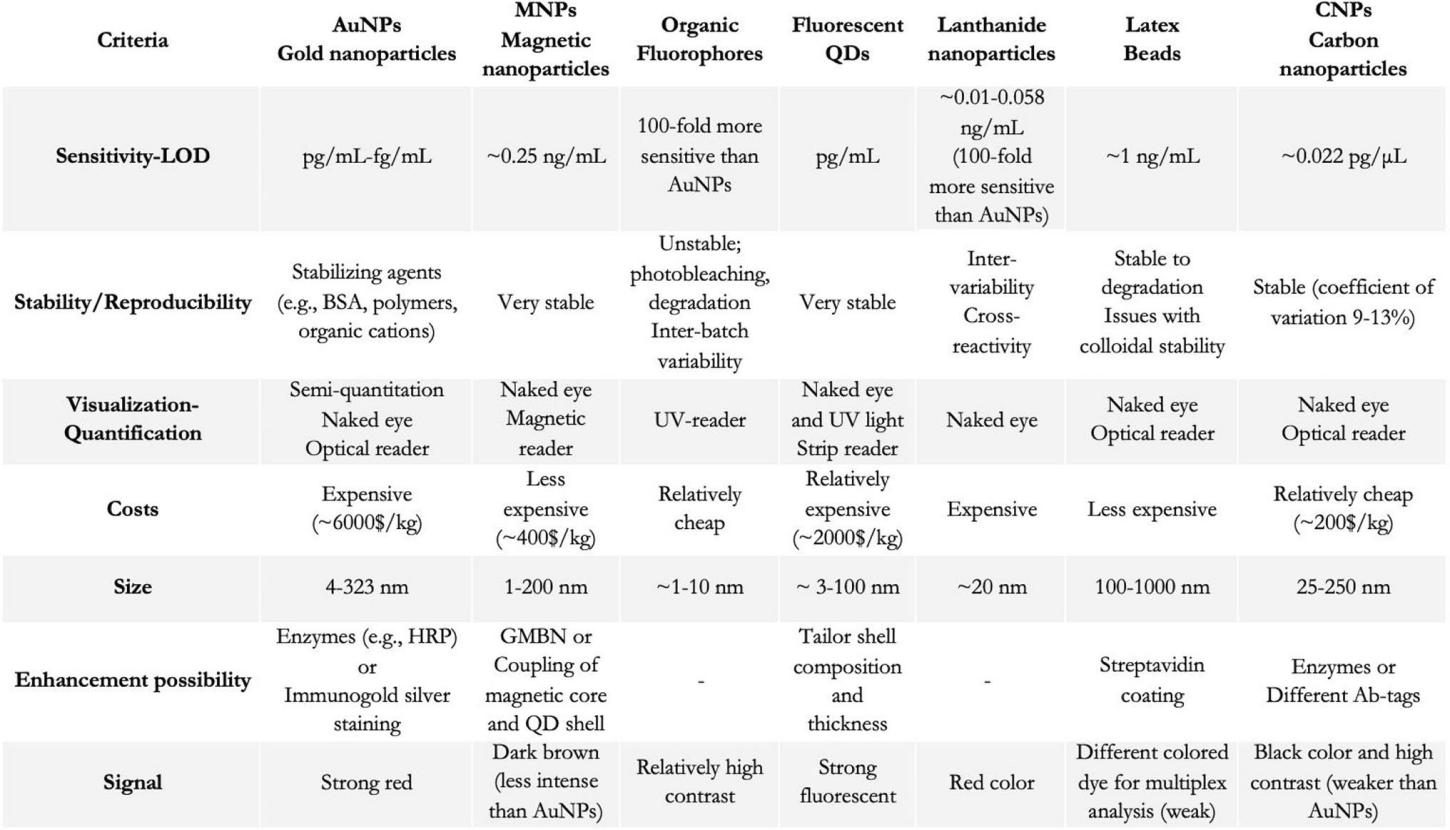
Overview of the state-of-the-art visual enhancers with focus on their relevant characteristics for the forensic field

Moreover, the versatility of CNPs is restricted compared to other nanoparticles. For instance, DNA oligonucleotide sequences can directly bind to the surface of AuNPs via thiol linkages, a process not feasible with CNPs. While methods exist for specific targeting in biochemical systems, they are generally not as straightforward or reliable as with AuNPs. For instance, the conjugation of additional immunoglobulins to CNPs that specifically target DNA molecules would be necessary, thereby further increasing production costs [94]. In addition, concerns over the biocompatibility of the CNPs can arise, particularly for in vivo applications. Soot or carbon particles can induce inflammatory or toxic responses in biological systems. Therefore, this renders CNPs less attractive for medical applications [108, 109].

Nevertheless, some considerations must be made before proceeding with the practical implementation of LFA in case work. Biological traces and fingermarks can be found on (non)porous surfaces. Upon exposure to air, biological traces undergo evaporation and dehydration. Therefore, an adequate method for collecting those traces and subsequently placing them on the sample pad of the LFA strip must be set. For this purpose, different solvents (e.g., methanol-trimethyl silyl chloride, chloroform, dichloromethane, methanol-formic acid) can be implemented for resuspending the dried/solidified traces and extracting the compounds [102, 110]. Similar to DNA collection, the traces can be single/double swabbed or the surface bearing the trace can be cut out and placed inside the solvent solutions [110, 111]. However, it should be noted that both swabbing and extraction of the traces are destructive processes; thus, no further analysis can be executed. Hence, it is noteworthy that all necessary analyses take place (e.g., the marks are visualized and photographed, samples partially collected for STR analysis and resubmissions) prior to any further manipulation and the LFA. Finally, the sample and buffer volumes may vary between cases. The overall sample volume for a sensitive analysis, guaranteeing the proper flow rate on the strip is approximately 45 μL [112]. Since the exact analyte concentrations in the sample volume are unknown, it is imperative to develop a sensitive LFA that covers a broad range of concentrations and demonstrates repeatability [102, 107].

**Scheme 1:**
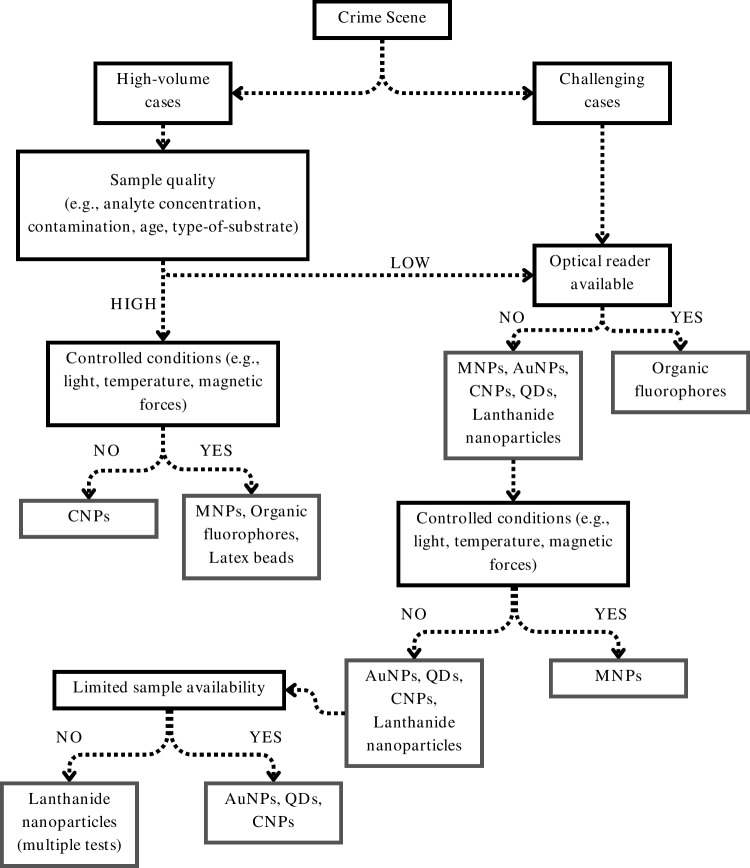
Suggested decision tree for selecting the visual enhancer to be implemented based on the specific case requirements

It is crucial to acknowledge the abovementioned challenges and consider the strict criteria for implementation in the forensic casework when selecting the most suitable LFA layout that is, a noncompetitive assay, and the visual enhancer (Scheme 1). The goal is to obtain the most sensitive, specific, yet simplistic, and fast trace analysis method while minimizing the costs. The results of this study may serve as a foundational point for subsequent research endeavors. However, other considerations must be made. The LFA consists of several components that can be individually optimized as to increase the overall sensitivity. For instance, the selection of an adequate mono- or poly-clonal antibody with optimal binding kinetics can significantly reduce the LOD and yield more accurate and reproducible results. Moreover, the optimization of the used buffers (i.e., blocking, loading, and extraction), the printing material (i.e., the nitrocellulose membrane and the pads), and the introduced sample volume must be further investigated [17]. It should be noted that all visual enhancers investigated in this review, with the exception of the organic fluorophores, can be visualized by the naked eye for a qualitative and semi-quantitative analysis. However, for the forensic application, accuracy and robustness are essential. Consequently, the coupling of the LFA strip to a portable strip reader for a more accurate and objective quantitation of the results is of added value. Furthermore, the accuracy and sensitivity of the LFA method can be compared to pre-established and validated techniques such as GC–MS [110].

## Conclusions

The chemical profiling of traces found at the crime scene can provide important intelligence information for the overall investigation. LFAs constitute portable, easy, cheap, and yet sensitive and specific methods of analysis that may provide important investigative leads. Since the traces can be analyzed directly at the crime scene, samples can be selected based on their relevance and quality. Here we review the most relevant visual enhancers to be used in a LFA setup for forensic casework. Based on the sensitivity, reproducibility, and applicability requirements, the most suitable combination for forensic application is identified. From high-volume crimes to more challenging cases, the use of a noncompetitive LFA where CNPs are used as enhancers can best adhere to these criteria, with sensitivity comparable to more expensive alternatives (e.g., QDs and AuNPs). As the field navigates towards more sustainable synthesis routes for QDs that will reduce the toxicity and environmental risks, carbon can be applied in most forensic scenarios (Scheme 1). The CNPs show great promise for signaling even the lowest amounts of analyte present in complex biological traces typically encountered at the scene. Future studies evaluating the performance of the proposed setup prior to case implementation are recommended. Moreover, the performance of this CNPs-LFA can be compared to that of other implemented enhancers such as QDs and AuNPs. The accuracy of the CNPs-LFA can be confirmed through other sophisticated and expensive analytical methods, namely, ELISA, GC–MS, and matrix-assisted-laser-desorption/ionization-mass spectrometry-imaging (MALDI-MSI).
